# Evaluation of clinical parameters influencing the development of bone metastasis in breast cancer

**DOI:** 10.1186/s12885-016-2345-7

**Published:** 2016-05-12

**Authors:** Joachim Diessner, Manfred Wischnewsky, Tanja Stüber, Roland Stein, Mathias Krockenberger, Sebastian Häusler, Wolfgang Janni, Rolf Kreienberg, Maria Blettner, Lukas Schwentner, Achim Wöckel, Catharina Bartmann

**Affiliations:** Department for Obstetrics and Gynecology, University of Würzburg Medical School, Josef-Schneider-Str. 4, 97080 Würzburg, Germany; Department for Obstetrics and Gynecology, University of Ulm Medical School, Prittwitzstr. 43, 89075 Ulm, Germany; Faculty of Mathematics and Computer Science, University of Bremen, Universitätsallee GW1, 28359 Bremen, Germany; Institut für Medizinische Biometrie, Epidemiologie und Informatik (IMBEI), University of Mainz, Obere Zahlbacher Str. 69, 55131 Mainz, Germany

**Keywords:** Breast cancer, Bone metastases, Skeleton, Breast cancer subtypes, BRENDA

## Abstract

**Background:**

The development of metastases is a negative prognostic parameter for the clinical outcome of breast cancer. Bone constitutes the first site of distant metastases for many affected women. The purpose of this retrospective multicentre study was to evaluate if and how different variables such as primary tumour stage, biological and histological subtype, age at primary diagnosis, tumour size, the number of affected lymph nodes as well as grading influence the development of bone-only metastases.

**Methods:**

This retrospective German multicentre study is based on the BRENDA collective and included 9625 patients with primary breast cancer recruited from 1992 to 2008. In this analysis, we investigated a subgroup of 226 patients with bone-only metastases. Association between bone-only relapse and clinico-pathological risk factors was assessed in multivariate models using the tree-building algorithms “exhausted CHAID (Chi-square Automatic Interaction Detectors)” and CART(Classification and Regression Tree), as well as radial basis function networks (RBF-net), feedforward multilayer perceptron networks (MLP) and logistic regression.

**Results:**

Multivariate analysis demonstrated that breast cancer subtypes have the strongest influence on the development of bone-only metastases (*χ*2 = 28). 29.9 % of patients with luminal A or luminal B (ABC-patients) and 11.4 % with triple negative BC (TNBC) or HER2-overexpressing tumours had bone-only metastases (*p* < 0.001). Five different mathematical models confirmed this correlation. The second important risk factor is the age at primary diagnosis. Moreover, BC subcategories influence the overall survival from date of metastatic disease of patients with bone-only metastases. Patients with bone-only metastases and TNBC (*p* < 0.001; HR = 7.47 (95 % CI: 3.52–15.87) or HER2 overexpressing BC (*p* = 0.007; HR = 3.04 (95 % CI: 1.36–6.80) have the worst outcome compared to patients with luminal A or luminal B tumours and bone-only metastases.

**Conclusion:**

The bottom line of different mathematical models is the prior importance of subcategories of breast cancer and the age at primary diagnosis for the appearance of osseous metastases. The primary tumour stage, histological subtype, tumour size, the number of affected lymph nodes, grading and NPI seem to have only a minor influence on the development of bone-only metastases.

## Background

Despite continual improvements achieved in the diagnosis and treatment of breast cancer (BC), 20 to 30 % of patients with early breast cancer will face relapse and develop potentially incurable distant metastases [1]. Therefore, the spread of malignant cells to distant sites and the growth of metastases is one of the most virulent attributes of cancer.

Despite extensive research on the spreading of tumour cells, a comprehensive understanding of the process of breast cancer metastases, including tumour cell seeding, tumour dormancy, and metastatic growth, is only partly understood. A better knowledge of the pattern of metastatic spread could help to adapt adjuvant therapies and to personalize follow-up examinations of cancer patients.

The existing molecular and immunological approaches of explanation for the spread of tumour cells and the formation of metastases focus on the vascular infiltration, circulation, epithelial adherence and extravasation of malignant cells. Moreover, the “seed and soil” hypothesis firstly published by Stephen Paget et al. plays a decisive role for description of the spread of tumor cells. This theory describes the organ-preference patterns of tumor metastasis as a product of favorable interactions between cancer cells and specific organ microenvironments [2, 3]. Considerable numbers of clinical studies underline the great interest in this subject [4, 5]. These explanatory models and clinical studies identified several correlations between breast cancer subtypes and clinical characteristics.

The bottom line of these studies is the unfavorable prognosis of tumours that are triple negative or that overexpress HER2. BC subtypes that express estrogen and progesterone receptors are correlated with a positive clinical outcome and the tendency to develop most likely osseous metastases [6–8].

Altogether, bone is the first site of distant disease in 25 to 40 % of women with advanced breast cancer. Although patients with osseous metastases have significantly better clinical outcome than women with visceral or cerebral metastases [9], bone constitutes a site of paramount importance for the development of distant metastases of breast cancer.

The establishment of metastases in the skeleton is based on mutual interactions of breast cancer cells with the osseous microenvironment consisting of osteoblasts and osteoclasts. The process of bone destruction and resorption and the release of growth factors by the last mentioned cells promote adherence, survival and proliferation of tumour cells. Therefore, bone destruction and growth of tumour cells constitutes a vicious circle [10]. Remodeling processes in the skeleton that take place at the time of early breast cancer development and dissemination could favour the growth of osseous metastases [11].

Considering these theories, several clinical and basic studies have been performed to find target factors associated with bone-specific distant recurrence of BC.

The International Breast Cancer Study Group analyzed recurrence date in a study population of 6000 patients, who were treated in seven adjuvant breast cancer trials in order to figure out patients at high risk for bone metastases [12]. Factors associated with increased rates of osseous recurrence included higher numbers of involved lymph nodes, larger tumour size and estrogen receptor (ER) expression. Lipton et al. tried to identify a subset of patients with breast cancer with a predilection to bone as the first site of distant recurrence by using a serum assay for the carboxyterminal peptide of type I collagen (CTx), a marker for bone turnover released during bone resorption [13].

Improving knowledge about the interaction of breast cancer cells and bone environment could help to determine and to define a subgroup of women and subtypes of breast cancer which have a high risk of developing osseous metastases. Moreover, these findings could help to develop personalized and tailored breast cancer therapy [13, 14].

In this retrospective study, we analysed the correlation between the risk for the development of bone-only metastases and different prognostic factors like primary tumour stage, the biological and histological subtype, the age at primary diagnosis, the tumour size, the number of affected lymph nodes as well as the grading. We were able to evaluate the importance of different clinical variables for the development of osseous metastases und resolve some apparent contradictions described in the literature.

## Methods

The comprehensive database BRENDA has been described in several publications [15, 16], and contains 886 patients with advanced breast cancer. The clinical data and information were collected between 1992 and 2008. Patients were diagnosed and treated at the Department of Gynecology and Obstetrics at the University of Ulm or in one of the 16 other certified breast cancer centers of the BRENDA-study group. The primary end point of this trial was the risk of the development of bone-only metastases and the prognostic impact of different variables like primary tumour stage, the biological and histological subtype, age at primary diagnosis, tumour size, the number of affected lymph nodes as well as the grading. Secondary end points were metastasis-free survival (MFS) with focus on bone as the only site of relapse and overall survival from date of advanced breast cancer. For each patient included in the study, a written consent form was obtained.

### Study cohort

The study population is based on a subgroup of patients of the BRENDA collective (*n* = 9625) comprising 886 women with evidence of distant metastases. The follow up was conducted for at least 10 years from date of primary diagnosis. In the study population of 886 women 226 (25.5 %) developed bone-only metastases within 10 years after primary diagnosis of breast cancer. Bone metastases was defined as morphological detection of metastases typical formations in the skeleton via medical imaging [17].

For the study cohort, primary tumour stage, the biological and histological subtype, the age at primary diagnosis, the tumour size, the number of affected lymph nodes, the Nottingham Prognostic Index (NIP) as well as the grading of the tumour were analyzed separately in relation to the risk of the appearance of bone-only metastases.

The TNM classification was used as published by the UICC to define the primary tumour stage. Secondly, the study cohort was split into two groups, women who were older than 65 years (>65 years) or younger than 65 years (≤65 years). In terms of histological subtypes, we set up three study groups: invasive ductal breast cancer, invasive lobular and others (comprising medullar, tubular and mucinous breast cancer subtypes).

To define the biological breast cancer subtypes, the cell proliferation marker Ki67 is currently used. As this marker was not determined for the BRENDA database we modified the St Gallen molecular subtypes as suggested by Parise et al., von Minckwitz et al. and Lips et al. We used the characteristics hormone receptor expression (HR), HER2 overexpression and tumour grade (low = tumour grade of 1 or 2; high = tumour grade of 3) instead: Luminal A is defined by HR positive, HER2 negative- and low tumor grade, luminal B HER2 negative (luminal B/HER-) by HR positive,HER2 negative and high tumor grade, whereas luminal B HER2 positive (luminal B/HER2+) represents HR positiveHER2 positive. The triple negative breast cancer (TNBC) is negative for HR and HER2. The HER2-overexpressing subtype is defined by negative HR and positive HER2 [18–21].

According to gene expression profiling (GEP), 71 % of triple-negative tumours showed a basal-like phenotype and 77 % of basal-like tumours showed a triple-negative phenotype. Basal-like cancers are a heterogeneous category comprising mainly infiltrating ductal carcinoma of no special type. Medullary, atypical medullary, metaplastic, secretory, myoepithelial, and adenoid cystic carcinomas of the breast also show a basal-like phenotype. The Nottingham prognostic Index (NPI) was calculated using the formula: NPI = [0.2 x S] + N + G. S is the size of the index lesion in cm, N is the nodal status: 0 nodes = 1, 1–3 nodes = 2, 4+ nodes = 3 and G is the grade of tumour: Grade I =1, Grade II =2, Grade III =3. Nottingham Prognostic Score (NPS) was calculated using NPI: NPI ≤ 3.4: low risk; NPI > 3.4 and ≤5.4: intermediate risk and NPI > 5.4: high risk. For classifying the grading of breast cancer, we applied the morphological assessment of the degree of differentiation of breast cancer described by Elston et al. [22]. Information on the time and site of first distant metastases was obtained from physicians responsible for follow-up care. Moreover, patients, as well as the local death registries, answered questionnaires.

### Statistical analysis

All categorical data were described using numbers and percentages. Comparisons of categorical variables between groups were made by using *χ*2 tests. Quantitative data were presented using median and range or mean and standard deviations. Overall survival from the time of metastases was defined as the interval between the first distant metastases and death. If the patient was lost to follow-up, data were censored at the date of the last known contact. When no information was available, the status was coded as missing data. Survival distributions and median survival times were estimated using the Kaplan–Meier product-limit method. The log-rank test was used to compare survival rates. Further, the Cox proportional hazards model was used to estimate the hazard ratio and confidence intervals. The proportional hazards assumption was assessed by including both the product of the individual terms and time in the models. To adjust for differing risk factor distributions between groups, the multivariate Cox proportional hazards regression models were used. Furthermore, we used two tree-building algorithms, “exhausted CHAID” (Chi-squared Automatic Interaction Detector) and CART (Classification and Regression Trees), with relapse to bone-only (yes or no) as the dependent variable and breast cancer subtype and other patient/tumour characteristics included as covariates. These associations were further examined in multivariate models using radial basis function networks (RBF-net), feedforward multilayer perceptron networks (MLP) and logistic regression. An RBF-network is an artificial neural network that uses radial basis functions as activation functions. The Bayesian Information Criterion (BIC) determines the number of units in the hidden layer. The "best" number of hidden units is the one that yields the smallest BIC in the training data. We used normalized radial basis functions as activation functions for the hidden layer, which "links" the units in a layer to the values of units in the succeeding layer. For the output layer, we used as activation function just the identity function; thus, the output units are simply weighted sums of the hidden units. The output of the network (bone-only metastases) is therefore a linear combination of radial basis functions of the inputs and neuron parameters. A multilayer perceptron (MLP) is a feedforward artificial neural network model that maps sets of input data onto a set of appropriate outputs (bone-only metastases). An MLP consists of multiple layers of nodes in a directed graph, with each layer fully connected to the next one. Except for the input nodes, each node is a processing element (neuron) with a nonlinear activation function. In our case, we used the hyperbolic tangent as activation function for the units in the hidden and output layer respectively. MLP utilizes backpropagation as supervised learning technique for training the network. To evaluate the performance of the models, we used receiver operating curves (ROC), as well as the predictiveness curve, a plot of cumulative percentage of individuals to the predicted risks.

Cumulative percentage indicates the percentage of individuals that have a predicted risk equal to or lower than the risk value. Statistical analyses were two-sided and p-values less than 0.05 were considered statistically significant. We used R-3.20, IBM SPSS 22 and RapidMiner 6.

## Results

### Characteristics of the study cohort

We probed a study population of 9625 female breast cancer patients. Our study cohort consisted of 886 (9.2 %) patients with confirmed metastatic breast cancer. 226 (25.5 %) women developed bone-only metastases within 10 years after primary diagnosis of breast cancer. The median age at primary diagnosis of the 226 patients with bone-only metastases was 67.0 years (y) [range: 30-93y] and at distant relapse 69y [range: 33-93y]. Nearly 50 % of the 226 (25.5 %) patients with bone-only metastases had a metastatic free survival of just 1 y [median 13 months; 95 % CI 7.4–18.6 months]. On the other side, the maximum metastatic free survival (MFS) was 16.4 y, but the percentage of patients with bone-only metastases and MFS > 10 years was very small (1.8 %). For patients with other sites of relapse, the maximum MFS was 11.8 years (Table 1).

**Table 1:** Basic characteristics

patients with advanced breast cancer		bone-only metastases			*p*-value
		Total	yes	no	
		886	226 (25.5 %)	660 (74.5 %)	
Age at primary diagnosis (in years)		mean: 61 (SD 14.2) (median:62)	mean: 65 (SD 14.3) (median:67)	mean: 60 (SD 13.9) (median: 61)	0.03
		Range: 22–96	Range: 30–93	Range:22–96	
Metastatic free survival (MFS) (in months)		mean: 25 (SD 27.7) (median:18)	mean: 23 (SD 33.3) (median:13)	mean: 26 (SD 25.4) (median: 19)	0.03
		Range: 0–197	Range: 0–197	Range:0–142	
T-categories (in absolut numbers (percent)	T1	283 (31.9)	72 (25.4)	211 (74.6)	0.385
	T2	485 (54.7)	118 (24.3)	367 (75.7)	
	T3/T4	118 (13.3)	36 (30.5)	82 (69.5)	
Menopausal status (dto.)	premenopausal	204 (23.0)	37 (18.1)	167 (81.9)	0.026
	perimenopausal	31 (3.5)	6 (19.4)	25 (80.6)	
	postmenopausal	649 (73.3)	182 (28.0)	467 (72.0)	
	unknown	2 (0.2)	1 (50.0)	1 (50.0)	
Receptor staus (dto.)	negative	210 (23.7)	24 (11.4)	186 (88.6)	<0.001
	positive or unknown	676 (76.3)	202 (29.9)	474 (70.1)	
HER2/neu (dto.)	negative or unknown	704 (79.5)	190 (27.0)	514 (73.0)	0.047
	positive	182 (20.5)	36 (19.8)	146 (80.2)	
Grading (dto.)	1	26 (2.9)	8 (30.8)	18 (69.2)	0.002
	2	416 (47.0)	127 (30.5)	289 (69.5)	
	3	444 (50.1)	91 (20.5)	353 (79.5)	
Nodal staus (dto.)	nodal negative	268	63 (23.5)	205 (76.5)	0.716
	1 < = N < = 3	198	49 (24.7)	149 (75.3)	
	3 < N < =10	198	49 (24.7)	149 (75.3)	
	N > 10	193	51 (26.4)	142 (73.6)	
sub-categories (dto.)	luminal A	352 (39.7)	115 (32.7)	237 (67.3)	<0.001
	luminal B/HER2-	221 (24.9)	62 (28.1)	159 (71.9)	
	luminal B/HER2+	103 (11.6)	25 (24.3)	78 (75.7)	
	TNBC	131 (14.8)	13 (9.9)	118 (90.1)	
	HER2-overexpressing	79 (8.9)	11 (13.9)	68 (86.1)	

### Univariate and multivariate analysis examining factors associated with bone-only-specific distant recurrence in breast cancer

We could identify a highly significant difference in bone-only metastases behaviour between invasive ductal and invasive lobular/other subtypes of breast cancer. 35 % of patients with lobular/other subtypes of breast cancer and 23 % with invasive ductal carcinoma had bone-only metastases (*p* = 0.002). There was no significant difference between lobular and other subtypes (*p* = 0.241).

**Fig. 1:**
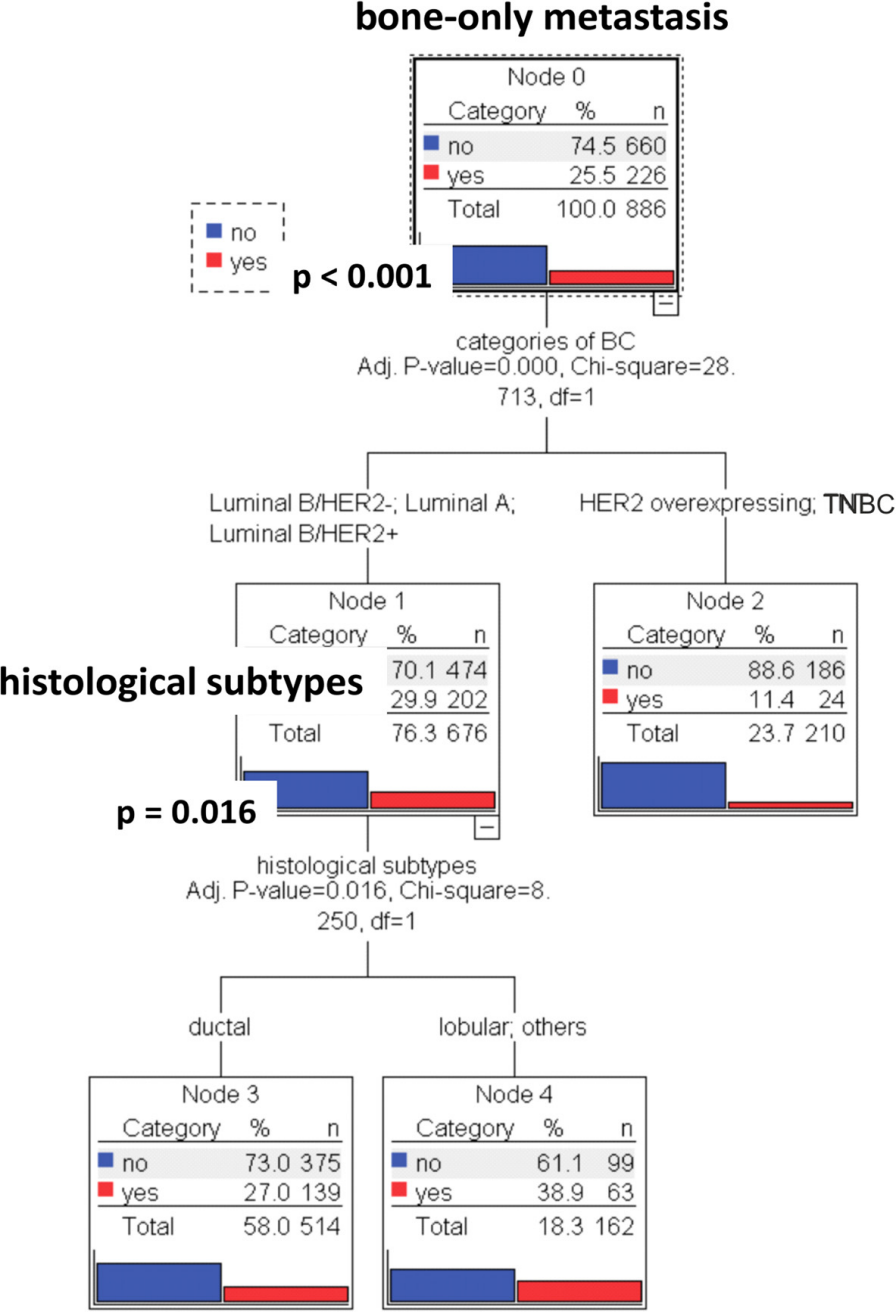
Multivariate Analysis (MA) demonstrates the strong interaction of BC subcategories with the dependent variable: bone-only metastasis. Independent variables: subcategories of BC and BC histological subtypes. Dependent Variable: Bone-only metastasis

In the next step, we analyzed whether the histological subtype of breast cancer is still significant for the development of bone-only metastases in a multivariate analysis integrating subclasses of BC and histological subtypes. This analysis revealed that breast cancer subtype has the strongest influence on the development of bone-only metastases (*χ*2 = 28). 29.9 % of patients with luminal A or luminal B and 11.4 % with TNBC or HER2-overexpressing tumours had bone-only metastases (*p* < 0.001). The histological subtype is decisive for patients with luminal A or luminal B (*χ*2 = 8). In this subclass, 27.0 % of patients with invasive ductal and 38.9 % with lobular/other carcinomas had bone-only metastases (*p* = 0.016) (Fig. 1). 10.3 % of the patients with TNBC or HER2-overexpressing invasive ductal carcinomas had bone-only metastases.

**Fig. 2:**
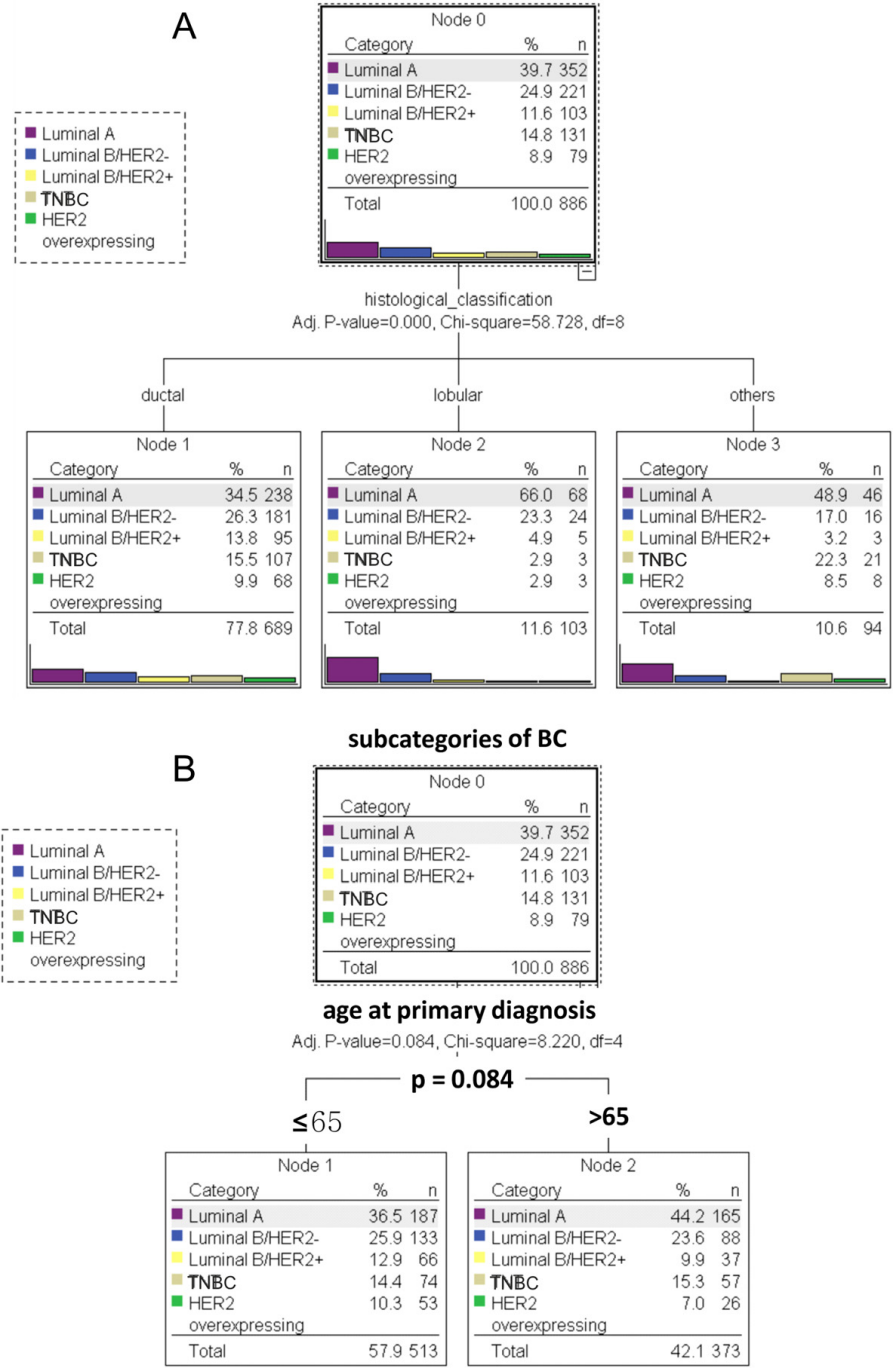
**a** Subcategories of BC classified by histological subtypes. The analysis demonstrates that BC histological subtypes are associated (*p* < 0.001) with distinct patterns of breast cancer subcategories. **b** Correlation of BC subcategories and age at primary diagnosis. Univariate analysis shows that age at primary diagnosis is a significant parameter for classifying bone-only histological subtypes (*p* = 0.001). Independent Variable: age at primary diagnosis. Dependent Variable: histological subtypes

In addition, there is a highly significant (*p* < 0.001) difference in tumour subclasses between various histological subtypes. 89.3 % of the patients with invasive lobular carcinoma and 60.8 % with invasive ductal carcinoma had luminal A or luminal B/HER2- tumours. Patients with the invasive lobular carcinoma had a significantly higher percentage of luminal A or luminal B/HER2- tumours compared to patients with ductal carcinoma (Fig. 2a).

Next, we analyzed the influence of age at date of primary diagnosis. Univariate analysis showed a highly significant difference in bone-only metastases behaviour between women younger than 65 years and women older than 65 years (*p* < 0.001). Only 20.1 % of women younger than 65 years developed bone-only metastases, whereas 33.0 % of patients older than 65 years suffered from bone-only metastases. Multivariate analysis together with subtypes of BC illustrated that age is the second strongest influence (*χ*2 = 17) after subtypes of BC (*χ*2 = 28). In the subtypes of patients with luminal A or luminal B BC, 23.6 % of patients younger than 65 and 38.3 % of patients older than 65 had bone-only metastases (*p* < 0.001). Tumour size and nodal status were no significant factors for bone-only metastases (both *p* = 1.0).

After age and histological subtype, we investigated the influence of tumour grading. Univariate analysis demonstrated a highly significant difference in bone-only metastases occurrences between patients with G3-tumours and G1 or G2-tumours (*p* = 0.001). 20.5 % of the patients with G3-tumours and 31.1 % of the patients with G1/G2-tumours had bone-only metastases. Applying multivariate analysis and integrating subclasses of BC demonstrates that subclasses are the only significant prognostic factors for the development of bone-only metastases. A partial explanation for this result is given by the fact, that grading is part of the definition of subclasses. 36.5 % of the G3-patients but only 10.6 % of the G1/G2-patients were TNBC or had a HER2 overexpression.

Further univariate analysis illustrated that the age at primary diagnosis is significantly correlated with the histological subtype of BC. 8.8 % (82.1 %) of patients younger than 65 years (≤65) and 15.5 % (71.8 %) of patients older than 65 years (>65 years) at primary diagnosis developed lobular (ductal) carcinoma (*p* = 0.001). However, there is no significant difference between subcategories of BC and age at primary diagnosis (*p* = 0.084) (Fig. 2b).

### Subcategories of breast cancer and age at primary diagnosis are both important independent variables for the development of bone-only metastases

**Fig. 3:**
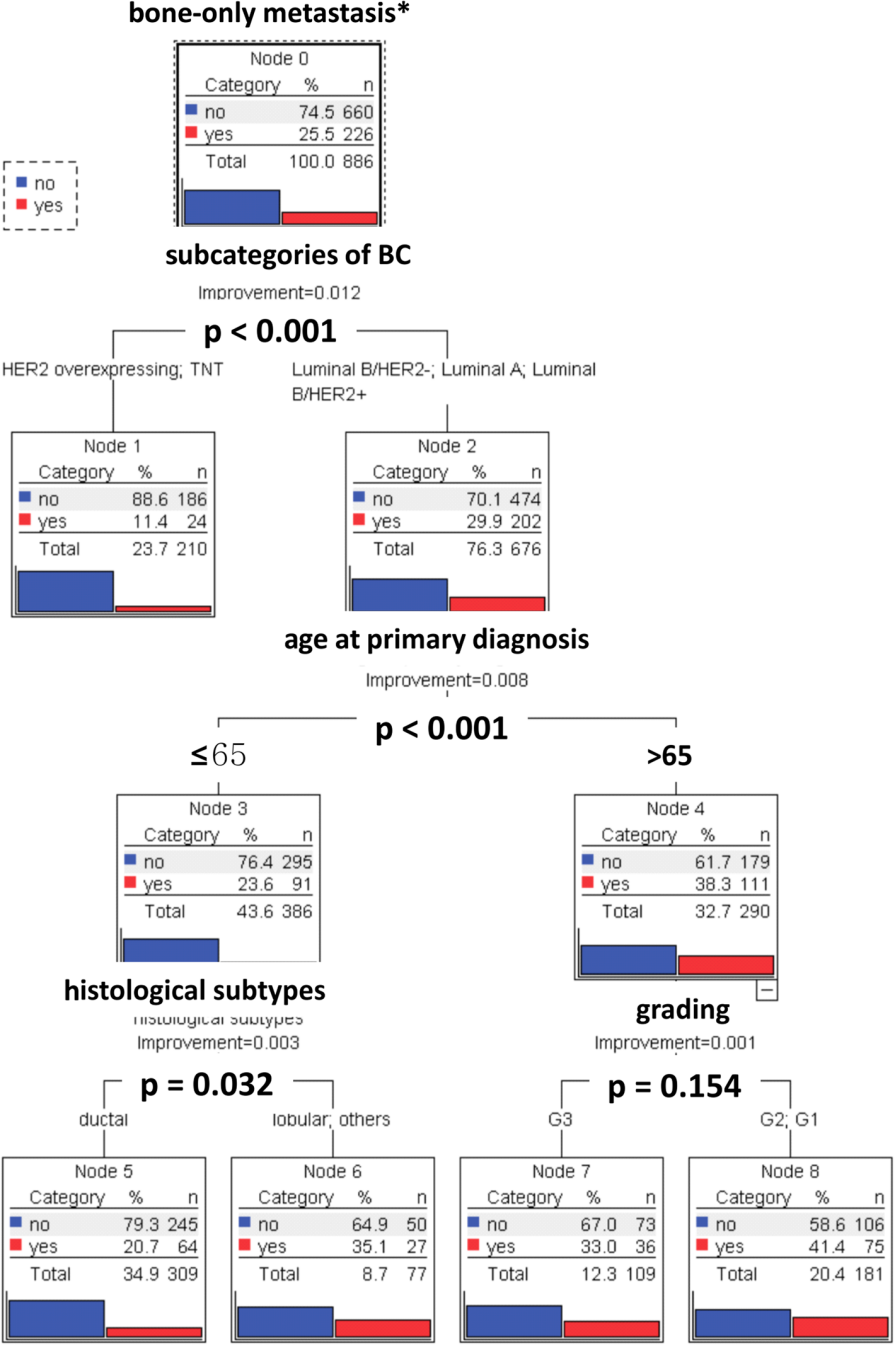
The decision tree derived by exhausted Multivariate Analysis (CART) illustrates that subcategories of BC, age at primary diagnosis, histological subtypes and grading are correlated with the appearance of bone-only metastases. Independent Variables: subcategories of BC, histological subtypes, age at primary diagnosis, tumor size, number of affected lymph nodes and grading. Dependent Variable: bone-only metastasis

Finally, we attempted to find out in a multivariate analysis, which of the following primary tumour factors (1) subcategories of BC (luminal A, luminal B/HER2-, luminal B/HER2+, TNBC and HER2 overexpressing), (2) histological subtypes, (3) age at primary diagnosis, (4) tumour size, (5) number of affected lymph nodes, (6) grading and (7) NPI are associated with bone-only-specific distant recurrence in BC. We compared the results of five different models: 1. Exhausted CHAID (decision-tree algorithm), 2. CART (classification and regression trees), 3. Radial Basis Function Network, 4. Multilayer Perceptron and 5. Logistic Regression. The decision-tree algorithms exhausted CHAID and CART revealed that the subcategories of BC, age at primary diagnosis and the histological subtypes were the three significant factors associated with bone-only-specific distant recurrence. Subcategories of BC has the strongest influence (*χ*2 = 28), followed by age (*χ*2 = 17) at primary diagnosis and histological subtypes (*χ*2 = 7). There is a highly significant difference (*p* < 0.001) between patients with TNBC or HER-overexpressing BC (11.4 % bone-only metastases) and patients with luminal A or luminal B BC (29.9 % bone-only metastases). However, there is no significant difference between the subgroups luminal A, luminal B/HER2+ or luminal B/HER2- (*p* = 0.395). Age has the second strongest influence. In the subgroup of luminal A or luminal B BC, 38.3 % of the patients older than 65 years and 23.6 % younger than 65 years had bone-only metastases (*p* < 0.001). In contrast to this, neither age nor histological subtype were significant factors for TNBC or HER2-overexpressing BC. In the subgroup of luminal A or luminal B-patients who are younger than 65 years, we found a significant difference (*p* = 0.032) between patients with invasive lobular/other (35.1 % bone-only metastases) and invasive ductal carcinoma (20.7 % bone-only metastases). If these patients were older than 65 years, CART revealed grading as an additional important factor. In the subgroup of luminal A or luminal B patients older than 65 years, 41.4 % of the patients with G1/G2 tumours and 33.0 % with G3 tumour had bone-only metastases, but this result was no longer significant (*p* = 0.154; Bonferroni adjusted chi-square test) (Fig. 3).

Next, we used two data mining algorithms: Radial basis function (RBF) network and multilayer perceptron (MLP). We calculated and compared two different risk models: Risk model I included age at primary diagnosis, tumour size, the number of affected lymph nodes, grading, the subcategories of breast cancer, the histological subtypes and NPI. In contrast, risk model II consisted of the subcategories of breast cancer and the age at primary diagnosis. Analysis demonstrated that there was no significant difference between both risk models.

**Fig. 4:**
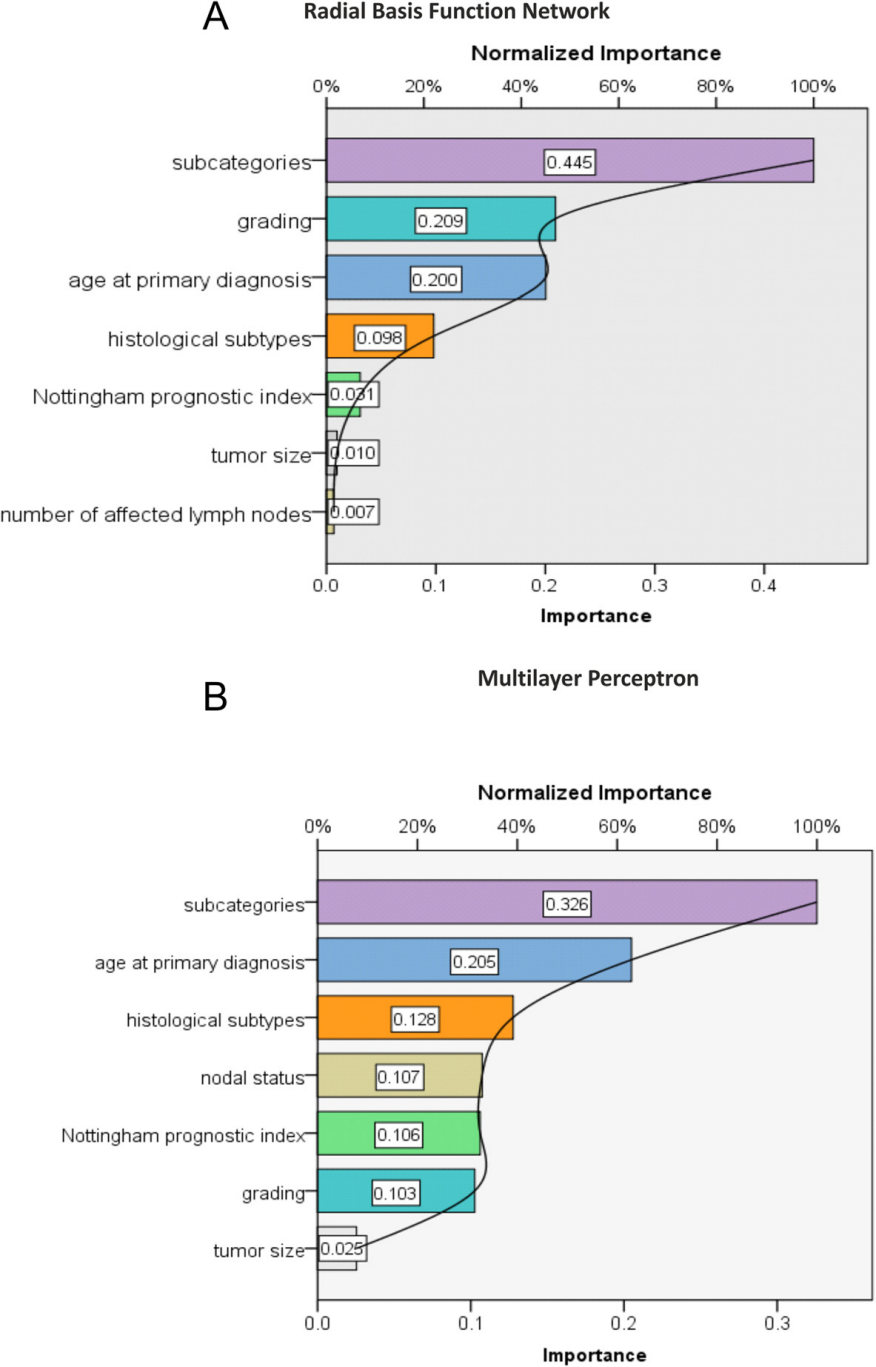
**a** Radial Basis Function Network (RBF) demonstrates that subcategories of BC and age at primary diagnosis are the most important independent variables for development of bone-only metastasis. The normalized importance of subcategories of BC is 100 % and of age at primary diagnosis 45 %. Independent Variables: age at primary diagnosis, tumor size, nodal status, grading, subcategories of BC, histological subtypes, Nottingham prognostic index. Dependent Variable: bone-only metastasis. **b** Multilayer Perceptron (MP) also demonstrates that subcategories of BC and age at primary diagnosis are the most important independent variables for development of bone-only metastasis. The normalized importance of subcategories of BC is 100 % and of age at primary diagnosis 62.9 %. Independent Variables: age at primary diagnosis, tumor size, nodal status, grading, subcategories of BC, histological subtypes, Nottingham prognostic index. Dependent Variable: bone-only metastasis

Subcategories of breast cancer and age at primary diagnosis were both important independent variables. The normalized importance of subcategories of breast cancer was 100 % for RBF and MLP. For the age at primary diagnosis, the normalized importance was 49.7 % for MLP and 45.9 % for RBF. The other variables seemed to have only a minor influence on the development of bone metastases. The area under the curve of the function generated by the RBF network was 0.64 and 0.66 in the case of MLP (Fig. 4a and b).

**Fig. 5:**
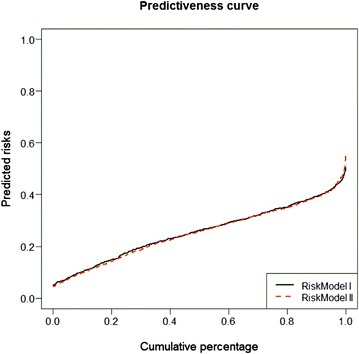
Logistic Regression Models (LRG) describes the relevance of different variables for the development of bone-only metastases: black line: risk model I, independent variables: Age at primary diagnosis, subcategories of breast cancer red line: risk model II, independent variables: Age at primary diagnosis, subcategories of breast cancer, tumour size, nodal status, grading, Nottingham prognostic index. The Predictiveness Curve is a plot of cumulative percentage of individuals to the predicted risks [42]

In a further analysis, we calculated and compared two binary logistic models as probabilistic classification models to predict bone-only-specific distant recurrence based on the same predictors as above. We could not, again, find a significant difference between risk model I and risk model II. Although risk model I contained the additional variables: tumour size, number of affected lymph nodes, grading, histological subtypes and NPI, subcategories of breast cancer and age at primary diagnosis were again the only important independent variables (Fig. 5).

Altogether, the five different models showed that the subclasses of BC and the age at primary diagnosis were the most important prognostic factors for bone-only metastases. Tumour size and nodal status played no significant part.

### Subcategories of breast cancer are decisive parameters for overall survival in patients with bone only analysis

**Fig. 6:**
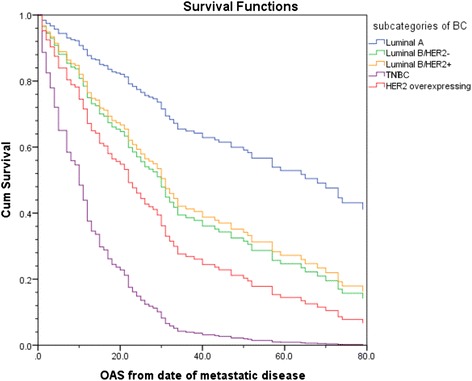
Overall Survival (OS) from date of metastatic disease of patients with bone-only metastases stratified by breast cancer subcategories. blue line: Luminal A, green line: Luminal B/HER2 negative, yellow line: LuminalB/HER2 positive, violet line: HER2 overexpressing/hormone receptor negative, red line: TNBC

Having clarified the importance of different variables for the development of bone-only metastases, we analyzed in a further step the effect of breast cancer subcategories on overall survival from date of metastatic disease in patients with bone-only metastases. Data were adjusted by age. Cox Regression showed that luminal B/HER2+ and luminal B/HER2- subcategories had a significantly worse OAS from date of metastatic disease compared to patients with luminal A BC (Fig. 6). Patients with TNBC (*p* < 0.001; HR = 7.47 (95 % CI: 3.52–15.87) and HER2 overexpressing BC (*p* = 0.007; HR = 3.04 (95 % CI: 1.36–6.80) had the worst outcome of patients with bone-only metastases.

### The development of bone-only metastases has changed in a period of almost 20 years

In the final analysis, we focused on the risk of developing bone-only metastases over a period of almost 20 years. For this, we divided patients in two cohorts for the decades 1992–2000 and 2001–2008 and analysed the risk of the appearance of bone-only metastases separately. The year 2000 was selected as intersection point as it coincides with the introduction of new therapy standards. Univariate analysis demonstrated that the year of primary diagnosis was a significant parameter for classifying bone-only metastases behaviour. 18.0 % of patients with primary diagnosis in 1992–2000 and 28.4 % with primary diagnosis in 2001–2008 had bone-only metastases (*p* = 0.001).

## Discussion

Breast cancer with bone-only metastases is usually thought to be associated with a relatively favourable prognosis with median survival times in the range of 24–36 months compared with breast cancer with both bone and other metastases or with non-bone sites of relapse [13]. This result was already confirmed in one of our previous studies: We had a median survival of 36.0 months [95 % CI 26.2–45.8] [23]. Therefore, the primary aim of the current study was to evaluate clinico-pathological risk factors as possible prognostic factors for the development of bone-only metastases. Univariate analysis showed highly significant interactions between bone-only metastases and histological subtypes, subcategories of BC, age and grading. Tumour size and number of affected lymph nodes had no significant interactions.

Whereas the clinical outcome of patients affected by breast cancer and bone metastasis has already been examined in several clinical trials [24, 25], other studies focus on the risk factors for the development of bone metastases. We could confirm the results published by James et al., showing that bone metastases were significantly more often associated with lower grade primary BC than less differentiated tumours [26]. First site metastases in the bone is more likely with lobular carcinoma than with the invasive ductal carcinoma (NST). This is consistent with the results of Purushotham et al. [27]. The same authors showed a relationship between increasing age at diagnosis and a reduction in risk of distant metastases to bone and viscera. In our trial, the results were just the opposite. BC patients older than 65 years developed bone-only metastases 1.5 times more often than younger BC patients did. Of course, our study was restricted to examining bone-only metastases.

One tool for estimating the risk of metastases is the Nottingham Prognostic Index (NPI), as described above. NPI is calculated using the tumour size, number of involved lymph nodes and grading. Since tumour size and number of involved lymph nodes had no significant relation to bone-only metastases, grade was the only remaining factor. As bone metastases are significantly more common in lower grade primary BC, patients with low risk NPI should have a higher percentage of bone-only metastases compared to patients with intermediate or high risk [17]. Indeed, 31 % of the low risk and 25 % of the intermediate or high risk patients had bone-only metastases, but this result was not significant.

Many publications describe the patterns of metastases of BC. The bottom line of these studies is the high risk of bone metastases for luminal A, luminal B/HER2+ or luminal B/HER2- tumours. TNBC, basal like and HER2 positive tumours tend however, to develop visceral and cerebral metastases [28–30]. These observations are in accordance with our achieved results. However, our analysis goes beyond these univariate correlations. We were able to determine the importance of these different variables for the development of bone-only metastases in several multivariate analyses. There was a highly significant correlation between subcategories of BC and bone-only metastases. Only 11 % of ABC-patients with triple negative or HER2-overexpressing tumours, but 30 % of ABC patients with luminal A and luminal B had bone-only metastases. Moreover, we could illuminate the importance of the histological subtype of BC and its influence on bone-only metastases, which is discussed contrarily in the literature. Dixon et al. and Korhonen et al. could not detect a significant difference between invasive lobular and invasive ductal BC and the frequency of bone-only metastases [31, 32]. Ferlicot et al., Jain et al. and Purushotham et al. however demonstrated a higher frequency of primary appearance of osseous metastases for the subgroup of invasive lobular BC [27, 33, 34].

We could resolve this apparent contradiction by using univariate and multivariate analysis. For univariate analysis, the histological subtype seems to be decisive. First, histological subtypes were linked to distinct patterns of breast cancer subcategories. 94 % of the patients with metastasized lobular invasive carcinoma, but only 75 % of the patients with ductal invasive carcinoma, had luminal A or luminal B subtypes.

The triple negative or HER2 positive breast cancer subtype, associated with a negative clinical outcome [15], appeared to be rare among the invasive lobular breast cancer subgroup. In contrast, 16 % of triple negative and 10 % of HER2 positive BC belong to the group of invasive ductal carcinomas. This finding is in accordance with the higher percentage of bone-only metastases in the subgroup of luminal A, luminal B/HER2 -, luminal B/HER2+ tumours as bone metastases is related to a better overall and recurrence free survival than other sites of metastases [35].

Secondly, taking all clinico-pathological risk factors in a multivariate model into consideration, subcategories of BC were the most important variable followed by age at primary diagnosis. 38 % of luminal A or B patients over 65 years had bone-only metastases. Histological subtypes were no longer a significant prognostic factor in this multivariate model.

In contrast to the significant correlation between histological subtypes and subcategories of BC, we were unable to find a significant correlation between BC subcategories and age at primary diagnosis; neither in the subgroups of patients with bone-only metastases nor in the whole group of BC-patients. This appears surprising, as the patterns of subcategories are significantly different with respect to age for the whole group of BC-patients.

For example, the percentage of patients with triple negative or HER2-overexpressing BC decreased with age: at primary diagnosis, 24 % of patients ≤35 years, 17 % between 35 and 65 years and 12 % >65 years had a TNBC or HER2-overexpressing BC. These findings indicate that biological mechanisms and biological subcategories influence the development of metastases in general and bone-only metastases in particular. This was independent of the age at primary diagnosis and needs to be further investigated.

To define the question of the risk factors for bone metastases, we calculated and compared risks models; one with all clinico-pathological factors and one with only subcategories and age. We used three different algorithms: there were no significant differences between the two models, independently of the algorithm used. Subcategories of breast cancer and age at primary diagnosis seemed to be the most important risk factors for developing bone-only metastases.

It is well known, that the risk of metastases development increases with the presence of lymph-node metastases, a larger sized primary tumour and loss of histopathological differentiation (grade), which are the established breast cancer prognostic markers [35]. None of these prognostic parameters seemed to have an influence on the development of bone-only metastases.

Finally, we investigated the effect of BC subcategories and age on overall survival from date of distant relapse for patients with bone-only metastases. Many publications demonstrate the prognostic effect in terms of overall and progression free survival for different subcategories of breast cancer in general [29, 30, 36]. Our analysis showed similar results. Luminal A patients with bone-only metastases had the best overall survival, while patients with TNBC or HER2-overexpressing BC had the worst OAS.

The development of bone-only metastases in the time period 2001–2008 compared to 1992–2000 seemed to be surprising at first sight. There was a significantly higher percentage of bone-only metastases in 2001–2008 compared to 1992–2000, although, due to new substances that reduce bone turnover [14, 37], we would have expected a constant or even lower percentage of bone-only metastases in the period 2000–2008. The higher percentage of patients with bone-only metastases could be explained by the growing number of older women with metastasized breast cancer in the second time period [38]. We demonstrated that patients tend to develop bone-only metastases more frequently with increasing age.

One limitation of this retrospective analysis is given by the fact that the study group was recruited between 1992 and 2008. The risk of recurrence as well as the patterns of metastases might have changed as therapeutic regimens have been modified and improved since then. In the nineties, only about 60 % of hormone receptor positive patients were treated with an anti-hormonal therapy. Aromatase inhibitors were not included in therapy of postmenopausal women. Moreover, the market approval of trastuzumab, pertuzumab and T-DM1 has additionally improved the outcome for patients with HER2 positive breast cancer [39, 40]. In terms of chemotherapeutic therapies, the discovery of paclitaxel as well as the application of intensive dose-dense chemotherapeutic regimes lead to a better outcome and has maybe affected the patterns of relapse [41].

Another limitation of this study might be undetected subclinical metastases during staging and screening investigations conducted within the framework of tumour after-care. On the other side, this statistical error affects each breast cancer subtype equally. Moreover, tumour follow up is based on clinical examinations. This approach has been conducted over the last 25 years and proven effective.

A decisive advantage of this study is the comprehensive documentation of every diagnosed metastatic lesion and, therefore, a precise description of tumour follow up.

## Conclusion

The bottom line of this analysis is the increasing risk for the development of metastases in the presence of lymph-node metastases, a larger sized primary tumour and loss of histopathological differentiation (grade). These factors are the established breast cancer prognostic markers. None of these prognostic parameters seem to have a dominant influence on the development of bone-only metastases. Subcategories of breast cancer and age at primary diagnosis were the only important parameters for the appearance of bone-only metastases in patients with advanced breast cancer. These findings can help to personalize and individualize adjuvant therapy and the tumour follow up examinations. Further research in this field is necessary.

### Availability of data and materials

Data will not be shared at the moment since these data are used for further publications.

### Ethical approval

The Ethics Committee of the University of Ulm, which covers all participating breast cancer centers of the BRENDA network, has approved this study and the BRENDA project.
